# Associations between the monocyte‑lymphocyte ratio and age‑related macular degeneration among US adults: evidence from NHANES 2005–2008

**DOI:** 10.1186/s40942-025-00766-2

**Published:** 2025-11-28

**Authors:** Zhanhe Zhang, Hongli Yang, Liangzhang Tan, Yongtao Li, Xinjun Ren, Xiaorong Li

**Affiliations:** Tianjin Key Laboratory of Retinal Functions and Diseases, Tianjin Branch of National Clinical Research Center for Ocular Disease, Eye Institute and School of Optometry, Tianjin Medical University Eye Hospital, No.251, Fukang Road, Tianjin, 300384 China

**Keywords:** Monocyte-lymphocyte ratio, Age-related macular degeneration, Blood cells, NHANES

## Abstract

**Background:**

Previous studies have established an association between age-related macular degeneration (AMD) and chronic systemic inflammation. However, the relationship between AMD and the monocyte-to-lymphocyte ratio (MLR), a novel inflammatory biomarker, remains unclear. In this study, we aimed to investigate the association between MLR and AMD using data from the 2005–2008 National Health and Nutrition Examination Survey (NHANES).

**Methods:**

Data from three NHANES cycles (2005–2008) were analyzed to preliminarily assess the association between MLR and AMD, excluding participants with incomplete data. We utilized weighted logistic regression models, restricted cubic spline functions (RCS) and constructed receiver operating characteristic (ROC) curves to evaluate the association between MLR and AMD.

**Results:**

A total of 4,894 participants were deemed eligible for our analysis, with 379 individuals diagnosed with AMD. The Monocyte to Lymphocyte Ratio (MLR) was significantly elevated in the AMD group compared to the non-AMD group. After adjusting for potential confounding factors, we found that elevated MLR levels were significantly associated with an increased risk of AMD, with an OR of 2.56, 95% CI: (1.17,5.58), *P* = 0.022. The restricted cubic spline (RCS) analysis revealed a significant nonlinear relationship between MLR and AMD, with an inflection point at 0.26 (nonlinear *P* < 0.05). Furthermore, the receiver operating characteristic (ROC) curve analysis demonstrated that MLR exhibited acceptable discrimination for AMD.

**Conclusions:**

Elevated MLR is associated with an increased risk of AMD, suggesting that MLR may serve as a simple and effective clinical biomarker of AMD.

## Background

Age-related macular degeneration (AMD) is the leading cause of vision loss and blindness among older adults in industrialized nations [1]. It accounts for more than 50% of blindness cases among white individuals aged 40 years or older in the United States [2]. The early stage of AMD is characterized by drusen formation and pigmentation changes, whereas the advanced stage includes dry geographic atrophy (GA) and wet choroidal neovascularization (CNV). The incidence of AMD sharply increases with age, and as life expectancy continues to rise, a substantial increase in AMD-related vision impairment is expected [1, 3, 4], drawing considerable attention from the scientific and medical communities. The etiology of AMD remains unclear. In addition to age [1, 3, 5], AMD has been associated with race [5], smoking [6], low antioxidant levels [7], reduced physical activity [8], systemic inflammation [9], and genetic factors [10, 11], among other factors. Patients with AMD exhibit chronic low-grade inflammation, particularly localized inflammation in the eyes [12–14]. Chronic inflammation is linked to vitreous membrane abnormalities, retinal pigment epithelium or photoreceptor degeneration, and CNV and plays a critical role in AMD pathogenesis [15, 16]. Routine blood examinations, liver function tests, and C-reactive protein (CRP) measurements are commonly used to assess acute infections; however, they lack sensitivity in detecting chronic inflammatory states.

The monocyte-to-lymphocyte ratio (MLR), an emerging and promising inflammatory biomarker, is calculated as the ratio of monocytes to lymphocytes in blood samples [17]. This marker has been extensively investigated in various inflammation-associated diseases, including cancer, tuberculosis, and cardiovascular conditions, and has been recognized as an indicator of systemic inflammation [18–20]. An elevated MLR has been linked to poor prognosis and rapid disease progression in conditions such as acute kidney injury and hematomas following brain contusions [21, 22]. Prior studies have suggested a correlation between increased peripheral monocyte counts and a higher prevalence of early, intermediate, and advanced AMD [23]. MLR is hypothesized to play a crucial role in AMD onset and progression, making it a potentially valuable marker for this condition. This study aimed to evaluate the clinical and strength of association of MLR in AMD and the findings may have important implications for the early detection, treatment, and overall management of AMD.

## Methods

### Study population in the National Health and Nutrition Examination Survey (NHANES)

**Fig. 1:**
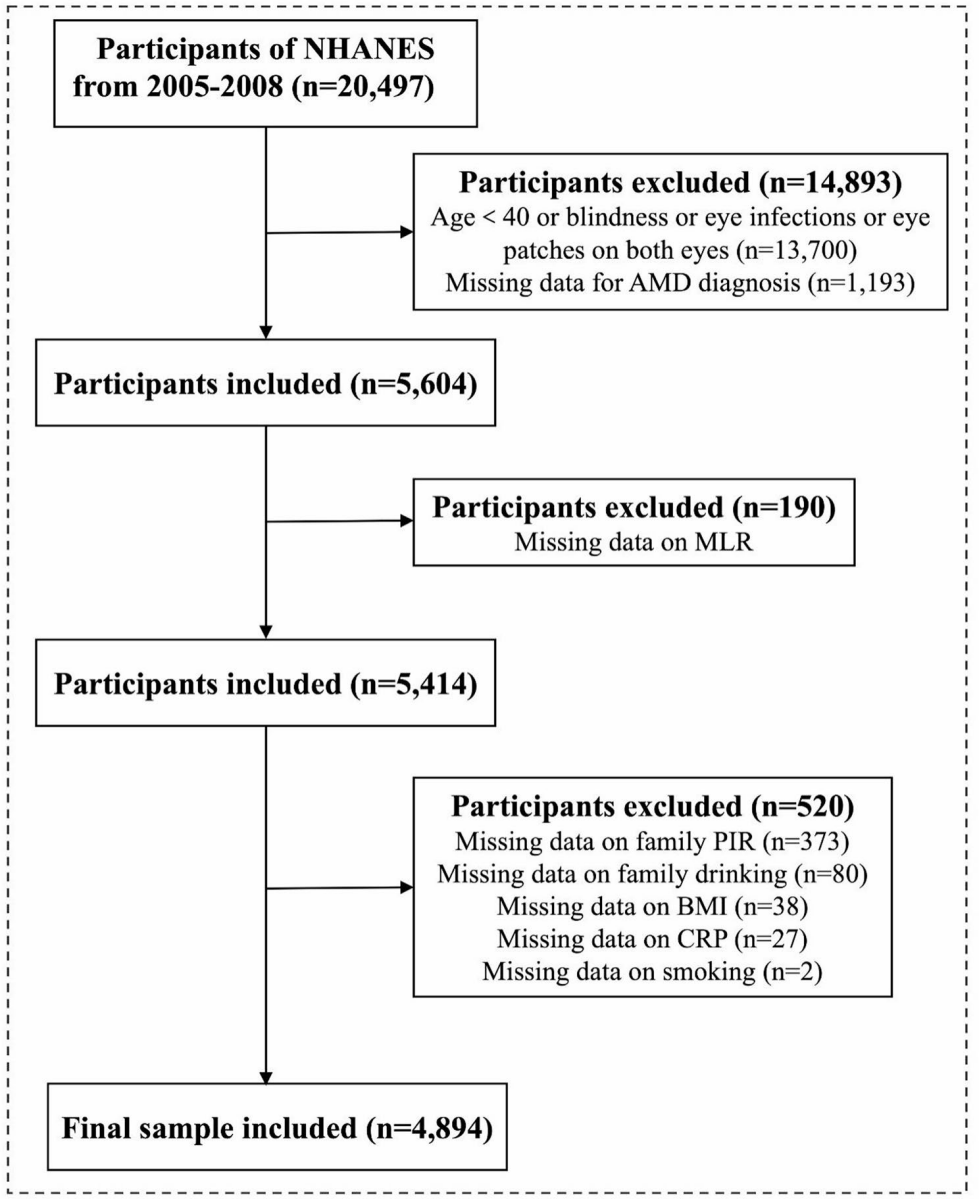
Graphic abstract of the study. Flowchart of sample selection from NHANES 2005–2008

The NHANES, conducted by the National Center for Health Statistics, evaluates the health and nutritional status of adult and pediatric populations in the United States. It employs a complex, multistage probability sampling design and surveys approximately 5,000 participants annually. Managed by the Centers for Disease Control and Prevention, NHANES ensures a demographically representative sample of the population in the United States through random selection. For this study, data from the 2005–2006 and 2007–2008 survey cycles were combined, as fundus imaging data related to AMD were available only for these cycles. Only individuals diagnosed with AMD based on fundus imaging were included. Participants were excluded if they had an eye patch, an eye infection, blindness, or were younger than 40 years. Those missing essential covariate data were also excluded. A total of 4,894 participants were included in the final analysis (Fig. 1).

### Definition and assessment of AMD and the MLR in NHANES

During the 2005–2008 NHANES survey, retinal photography was performed using the Canon CR6-45NM Ophthalmic Digital Imaging System with a Canon EOS 10D digital camera (Canon USA). Fundus photographs were graded by the University of Wisconsin–Madison using a modified version of the Wisconsin Age-Related Maculopathy Grading Classification Scheme 22. Early AMD was defined by the presence of drusen exceeding 500 μm in diameter, pigmentary disturbances, or both, whereas late-stage AMD was characterized by exudative changes or GA. Each image was assessed by at least two trained graders, with any discrepancies resolved by a third senior grader. If retinal images were available for both eyes, the eye with the more advanced condition was selected for analysis.

The MLR was calculated by dividing the absolute monocyte count by the absolute lymphocyte count, with both values obtained from laboratory data reports.

### Other covariates employed in NHANES

Given that other factors may have confounded the results, covariates were incorporated into the analysis. Drawing from NHANES interviews, laboratory tests, and questionnaires, variables were included, such as age, sex, race, education, poverty-to-income ratio (PIR), body mass index (BMI), smoking status, drinking status, CRP levels, and baseline health conditions, including hypertension and diabetes. Socioeconomic variables were assessed and recorded through household interviews. The PIR was categorized into two groups based on the original survey data: <2 and ≥ 2, with lower values indicating higher poverty levels. Participants’ behavioral characteristics were self-reported. The BMI was classified into three categories: <25 kg/m² (“underweight or normal weight”), 25–30 kg/m² (“overweight”), and ≥ 30 kg/m² (“obese”). Lifestyle factors included smoking status and drinking status. Drinking status was categorized as “Yes” or “No,” with individuals who had consumed fewer than 12 alcoholic beverages in their lifetime classified as never drinkers and all others categorized as drinkers. Smoking status [24] was classified into three groups: current smokers, former smokers, and never smokers. Participants who reported smoking at least 100 cigarettes in their lifetime and currently smoked daily or on some days were categorized as current smokers. Those who had smoked more than 100 cigarettes but no longer smoked were classified as former smokers, whereas individuals who had never smoked 100 cigarettes were classified as never smokers. Hypertension and diabetes were determined through self-reports of a physician’s diagnosis.

### Statistical analysis

DecisionLinnc1.0 software was used for NHANES data analysis. DecisionLinnc1.0 integrates multiple programming language environments and enables data processing, analysis, and machine learning through a visual interface. The study employed weighted sampling, which adjusted for any over- or under-representation in the survey data, allowing generalization to the population in the United States. Descriptive statistics were calculated for all participant pools. For continuous variables, the mean and standard deviation (SD) were reported based on data type, whereas categorical variables were presented as percentages. Chi-square tests were applied to categorical data, and t-tests were used for continuous variables (e.g., age). A logistic regression model was used to analyze the relationship between MLR and AMD, employing both unadjusted and multivariate-adjusted models: Model I (unadjusted), Model II (adjusted for sex, age, and race), and Model III (adjusted for all Model II covariates plus education, PIR, BMI, alcohol consumption, smoking, diabetes, hypertension, and CRP). To further investigate the relationship between MLR and AMD, restricted cubic splines (RCS) were employed, adjusting for all covariates in the spline model. The accuracy of MLR in diagnosing AMD was assessed using receiver operating characteristic (ROC) curves.

## Results

### Study population and baseline characteristics

Table 1 presents the baseline characteristics of the study population according to AMD status. A total of 4,894 participants, comprising 2,470 men (47.78%) and 2,424 women (52.22%), were included in the study. Among them, 379 unweighted participants had AMD. The predominant ethnic group was non-Hispanic white, with 2,632 participants (78.28%). No notable differences in BMI, diabetes, or CRP levels were observed between the non-AMD and AMD groups. However, significant differences were identified between participants with and without AMD in several variables, including age, race, education, smoking, drinking, family PIR, diabetes, hypertension, and MLR.

**Table 1:** Characteristics of included people based on AMD status in NHANES 2005–2008

Characteristic	Total (*n* = 4894)	No AMD (*n* = 4515)	AMD (*n* = 379)	*p* value
**CRP Mean (SD)**	0.44 (0.88)	0.44 (0.85)	0.55 (1.20)	0.066
**MLR Mean (SD)**	0.29 (0.12)	0.29 (0.12)	0.33 (0.19)	< 0.001
**Age (%)**				< 0.001
40–60	2500 (65.23)	2434 (67.80)	66 (27.78)	
60–85	2394 (34.77)	2081 (32.20)	313 (72.22)	
**Sex (%)**				0.800
female	2424 (52.22)	2242 (52.17)	182 (52.88)	
male	2470 (47.78)	2273 (47.83)	197 (47.12)	
**Race (%)**				0.002
Mexican American	751 (5.30)	705 (5.37)	46 (4.24)	
Non-Hispanic Black	957 (8.93)	923 (9.27)	34 (3.97)	
Non-Hispanic White	2710 (78.28)	2439 (77.71)	271 (86.61)	
Other Hispanic	321 (2.99)	303 (3.07)	18 (1.89)	
Other Race - Including Multi-Racial	155 (4.50)	145 (4.58)	10 (3.29)	
**Education (%)**				0.026
<=High school	2592 (43.58)	2378 (43.16)	214 (49.76)	
>High school	2302 (56.42)	2137 (56.84)	165 (50.24)	
**Smoking (%)**				< 0.001
Current	995 (20.52)	938 (20.89)	57 (15.20)	
Former	1587 (30.89)	1435 (30.22)	152 (40.74)	
Never	2312(48.58)	2142 (48.90)	170 (44.06)	
**Drinking (%)**				< 0.001
Yes	675(11.06)	602(10.60)	73 (17.67)	
No	4219(88.94)	3913(89.40)	306 (82.33)	
**Family-PIR (%)**				< 0.001
< 2	1986 (26.53)	1815 (26.00)	171 (34.15)	
>=2	2908 (73.47)	2700 (74.00)	208 (65.85)	
**BMI (%)**				0.078
obese	1863 (36.95)	1746 (37.36)	117 (31.02)	
overweight	1777 (35.51)	1622 (35.26)	155 (39.11)	
under/normal weight	1254 (27.54)	1147 (27.38)	107 (29.87)	
**Diabetes (%)**				0.390
No	4197 (89.74)	3868 (89.84)	329 (88.34)	
Yes	697 (10.26)	647 (10.16)	50 (11.66)	
**Hypertension (%)**				0.005
No	2670 (58.72)	2503 (59.48)	167 (47.64)	
Yes	2224 (41.28)	2012 (40.52)	212 (52.36)	

All proportions are weighted estimates of the US population characteristics, taking into account the complex sampling design of the National Health and Nutrition Examination Survey

All p values were calculated using t-test for continuous variables and the design-adjusted Rao-Scott Pearson χ2 test for categorical variables

AMD, age related macular degeneration ; CRP, C-reactive protein ; MLR, monocyte lymphocyte ratio ; PIR, Poverty–income ratio ; BMI, body mass index

### Positive association between MLR and AMD

Table 2 summarizes the findings of the logistic regression analysis examining the correlation between MLR and AMD. MLR was analyzed both as a continuous variable and as a categorical variable, categorized into quartiles (Q1–Q4), with various models incorporating different covariate adjustments. The data demonstrated a significant positive correlation between MLR and AMD risk, indicating that higher MLR levels were associated with a greater AMD risk. Specifically, in the fully adjusted Model 3, a one-unit increase in MLR corresponded to a 2.56-fold increase in AMD risk (95% CI: 1.17–5.58). When MLR was categorized into quartiles, AMD risk increased with higher quartiles (P for trend = 0.003 in unadjusted Model 1). However, this trend was not statistically significant in fully adjusted Model 3. Although quartile-based analysis of MLR did not show statistically significant differences across groups (Q2–Q4 vs. Q1), this may be due to non-linear effects, as suggested by the restricted cubic spline analysis. The continuous analysis remained significant, highlighting the importance of modeling MLR on a continuous scale.

**Table 2:** The association between MLR and AMD

	Model 1		Model 2		Model 3	
	OR(95%CI)	*p*-value	OR(95%CI)	*p*-value	OR(95%CI)	*p*-value
MLR	9.35 (4.09,21.40)	0.003	3.09(1.48,6.45)	0.004	2.56(1.17,5.58)	0.022
MLR(quartile)						
Q1	Ref		Ref		Ref	
Q2	1.27 (0.83,1.95)	0.25	1.08 (0.70,1.65)	0.73	1.06 (0.68,1.66)	0.77
Q3	1.33 (0.87,2.03)	0.18	1.00 (0.65,1.57)	0.98	0.99(0.61,1.59)	0.96
Q4	1.84 (1.21,2.81)	0.01	1.09 (0.73,1.64)	0.65	1.03 (0.67,1.57)	0.90
P for trend	0.003		0.72		0.98	

Model 1: no covariates were adjusted

Model 2: age, sex and race were adjusted

Model 3: fully-adjusted mode, which adjusts for age, sex, race, education, family-PIR, smoking, drinking, BMI, diabetes, hypertension, CRP

OR, odds ratio; 95% CI, 95% confidence interval

AMD, age related macular degeneration ; CRP, C-reactive protein ; MLR, monocyte lymphocyte ratio ; PIR, Poverty–income ratio ; BMI, body mass index

### Nonlinear associations between MLR and AMD

**Fig. 2:**
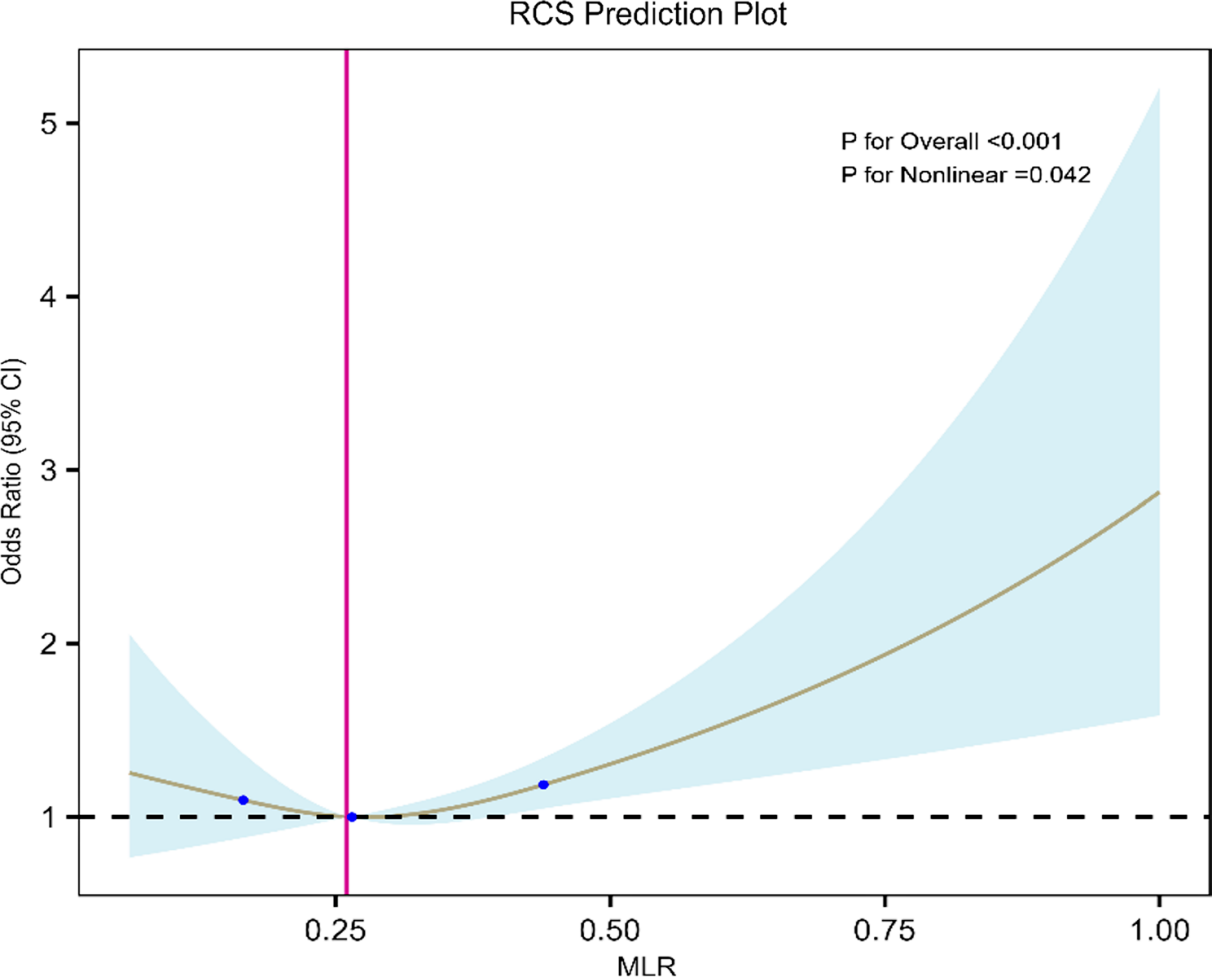
Non-linear relationship between MLR and AMD. The relationship between MLR and AMD in various groups was visualized using restricted cubic splines (RCS). The solid brown line represents the odds ratio (OR) for AMD in relation to the MLR, derived from the fully adjusted Model 3. The blue shaded area denotes the 95% confidence interval. The reference point is set at MLR = 0.26, corresponding to an OR of 1.00.

The RCS analysis (Fig. 2) revealed a J-shaped nonlinear association between MLR and AMD. The relationship demonstrated a gradual increase in AMD risk with rising MLR values, with a notable inflection point at approximately 0.26. Notably, even when MLR was below 0.26, the odds ratio (OR) remained above 1.00, indicating a persistently elevated AMD risk throughout the observed MLR range. Specifically, the estimated OR at MLR = 0.26 (reference point) was 1.00. Above 0.26, the risk increased more steeply, indicating a dose-dependent pattern characterized by relatively stable risk at lower MLR levels and a sharp elevation beyond this threshold, rather than reflecting a true protective effect.

### ROC curve analysis

**Fig. 3:**
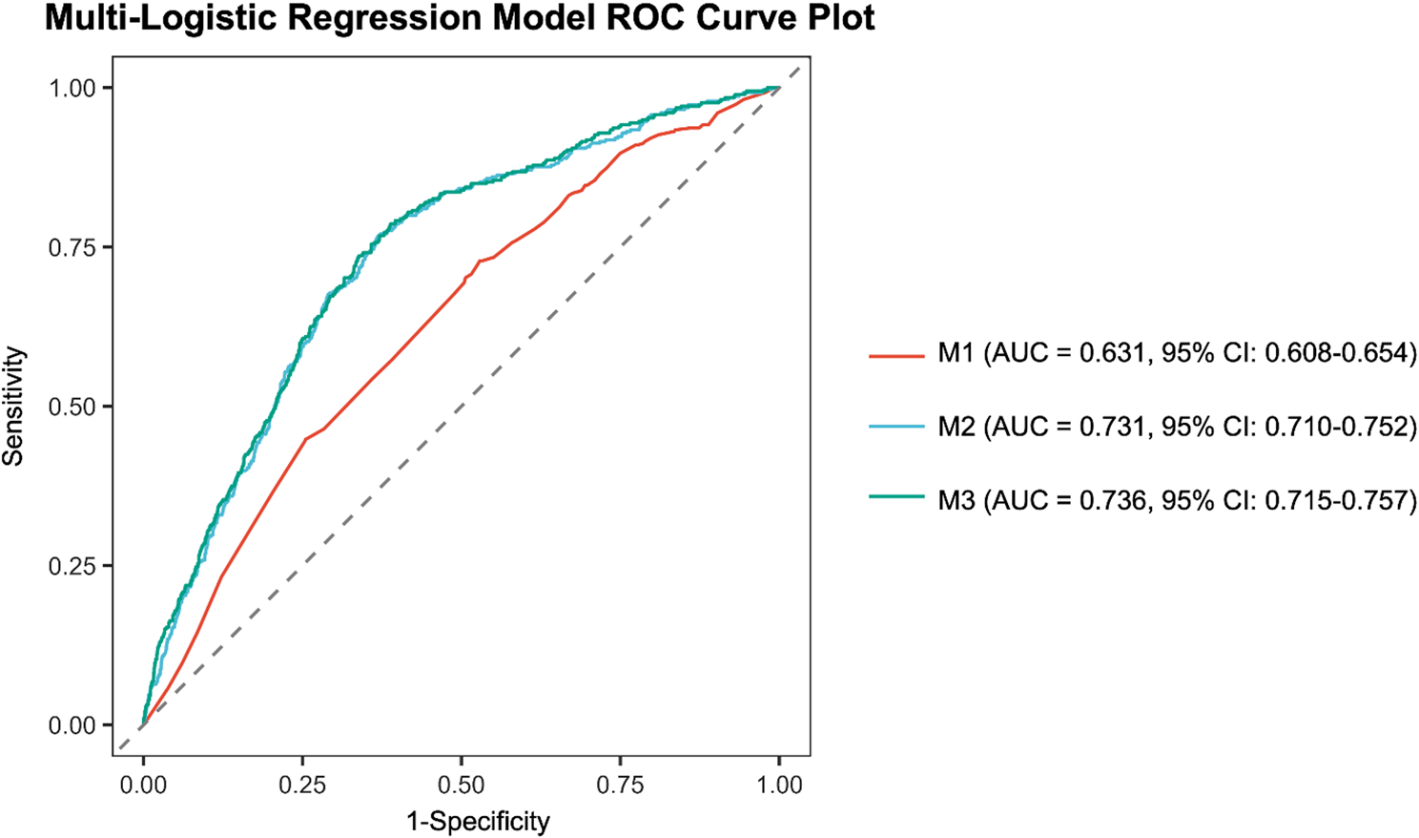
The receiver operating characteristic (ROC) curves for the Model-I ~ Model-III are presented. Model-I: age, sex, race, family-PIR and smoking were adjusted. Model-II: age, sex, race, family-PIR, smoking and MLR were adjusted. Model-III: maximum model, which adjusts for age, sex, race, education, family-PIR, smoking, drinking, BMI, diabetes, hypertension, CRP, MLR.

As shown in Fig. 3, the diagnostic model with the highest area under the curve (AUC = 0.736, 95% CI: 0.715–0.757) incorporated variables such as age, sex, race, BMI, family PIR, education, drinking, smoking, hypertension, diabetes, CRP, and MLR. However, this comprehensive model, referred to as Model III, included an excessive number of variables, limiting its practical applicability in clinical settings. To address this, efforts were made to identify a more streamlined diagnostic model with comparable accuracy. Upon evaluating models with five or fewer variables, a notable decline in discriminative performance was observed compared with Model III. When the number of variables was increased to six in Model II, the model (0.731, 95%CI 0.710–0.752) exhibited discriminative performance similar to Model III (AUC = 0.736, 95%CI: 0.715–0.757).These six variables included age, sex, race, family PIR, smoking, and MLR. The ROC curves for Models II and III closely aligned, and the z-test for AUC indicated no significant difference in discriminative performance between the two models for AMD (z = -1.34, *P* = 0.18). When MLR was removed from Model II to form Model I, a marked reduction in AUC was observed (0.631, 95% CI: 0.608–0.654 compared to 0.731, 95%CI: 0.710–0.752; z = -8.16, *P* < 0.001), underscoring the substantial contribution of MLR to the diagnostic accuracy of the AMD classification model.

## Discussion

Using NHANES data, this study demonstrates a significant association between elevated MLR and increased AMD incidence, even after adjusting for confounders, suggesting MLR as a potential biomarker associated with AMD. Moreover, the observed association highlights its potential utility in risk stratification for concurrent AMD. Whether early detection and intervention in patients with elevated MLR may help prevent or slow AMD progression remains to be determined by prospective studies, which could thereby improve patient outcomes and mitigate the impact of severe complications.

AMD is a complex, chronic, and progressive neurodegenerative disorder with a multifaceted etiology. Chronic inflammation and hypoxia contribute to oxidative stress and normal retinal aging. Persistent oxidative stress can lead to inflammation and tissue damage. The pathogenesis of AMD involves low-level chronic inflammation [15]. Subhi and Lykke Sørensen identified a correlation between systemic leukocyte activity and the early stages of wet-type AMD, linking this activity to lesion size and best-corrected visual acuity [25]. Systemic inflammation affects various blood cells, including neutrophils, lymphocytes, monocytes, and platelets, through fat metabolism and oxidative stress, which can damage islet cells and deplete nutrients [26]. Inflammation results from a complex interplay between immune cells, such as neutrophils, lymphocytes, and macrophages. Clinical studies have indicated that lymphocyte, neutrophil, and white blood cell counts, along with their ratios, may serve as indicators of chronic inflammation [27]. Ji et al. reported that MLR could reflect the circulating immune status of the host [28]. Compared with the individual counts of monocytes, lymphocytes, and leukocytes, MLR is considered more stable due to the balance between monocyte and lymphocyte levels, which is less susceptible to various physiological and pathological influences. As an easily accessible and cost-effective index derived from routine blood tests, MLR has emerged as a novel inflammatory biomarker, potentially providing valuable insights into the diverse clinical states of patients with AMD.

Ongoing research focuses on the impact of systemic inflammation on the potential mechanisms underlying both dry and wet forms of AMD [29]. In a study by Wu et al., an examination of inflammatory markers in patients with AMD compared to healthy individuals revealed no significant association between traditional inflammatory indicators, including high-sensitivity CRP, interleukin, and white cell count, and the presence of AMD [30]. Mine et al. observed differences in the MLR among patients with wet-type AMD, dry-type AMD, and healthy controls [31].

In vitro studies confirm that the accumulation of mononuclear phagocytes in the subretinal space is associated with photoreceptor loss [32]. Coughlin et al. demonstrated that uncontrolled complement-driven inflammation integrates lymphocytes into the pathogenesis of AMD and accelerates the pathological progression of CNV [33]. Some studies have elucidated the possible mechanisms by which monocytes and lymphocytes contribute to the pathogenesis of AMD. However, the exact relationship between the various inflammatory mechanisms and pathological progression remains unclear.

Considering the established role of MLR in various inflammatory conditions and the recognized involvement of chronic inflammation in AMD pathogenesis, we propose a potential mechanism for the observed elevation of MLR in AMD. Although this study did not directly evaluate biological mediators such as complement proteins or T cell subsets, previous literature provides a basis for hypothesis generation. Specifically, activation of the complement system (e.g., C3 and C5) and oxidative stress from light and oxygen exposure may initiate local and systemic inflammatory responses. These processes may promote monocyte-derived macrophage infiltration and the secretion of pro-inflammatory cytokines, which in turn activate T cells and perpetuate inflammatory signaling. Sustained immune activation could eventually lead to T cell exhaustion, contributing to a persistent imbalance between monocytes and lymphocytes, and thus an elevated MLR. This proposed pathway is speculative and warrants further mechanistic investigation in future studies.

This study had several strengths. First, by aggregating data from all available continuous NHANES cycles, we obtained a large, representative sample of the United States population. Second, rigorous control of confounding variables enhanced the credibility of our results. Third, to the best of our knowledge, this is the first study to investigate the association between MLR and AMD by integrating large-scale public databases, we employed multiple statistical methods to assess the relationship between MLR and AMD risk from different perspectives, strengthening the robustness and consistency of our findings.

However, this study has some limitations. First, reliance on the NHANES database and retrospective medical records introduced a cross-sectional study design, which precludes causal inference. Second, the long time span of the data may have introduced diagnostic inaccuracies. Given the imaging technology available during earlier periods and the combined classification of early and late AMD, some degree of underestimation or misclassification is possible, which may attenuate the observed associations. Third, the diagnosis of AMD in our study relied solely on fundus photography graded according to standardized protocols. While this method is well-established for AMD identification in large epidemiological studies like NHANES, it may not fully distinguish classic AMD from other pachychoroid spectrum diseases (e.g., central serous chorioretinopathy or polypoidal choroidal vasculopathy) which can present with similar fundoscopic features [34]. These conditions are hypothesized to involve distinct systemic inflammatory profiles, which may differ from classic AMD in their specific monocyte and lymphocyte activity [35–37]. Therefore, the potential inclusion of such cases could have acted as a confunder, influencing the observed association between MLR and AMD. Fourth, due to the limited number of cases, especially for late-stage AMD, we analyzed all AMD cases as a combined group. This approach precludes our ability to explore whether the strength of the association between MLR and AMD varies across different disease stages (early, intermediate, or late AMD) or subtypes (dry vs. wet AMD). It is plausible that the systemic inflammatory burden, as reflected by MLR, differs with disease progression. Finally, our analysis utilized data from the 2005–2008 NHANES cycles, as these were the most recent cycles that included both retinal fundus images and complete differential blood cell counts. While the large, nationally representative sample is a key strength, the imaging technology and diagnostic criteria from that era might differ from current standards. Despite these limitations, our findings establish a foundational association between MLR and AMD in a large population, and multicenter prospective studies are essential to validate these results and explore the underlying mechanisms.

## Conclusions

This study demonstrates a significant association between elevated MLR and AMD after accounting for confounding factors, reinforcing its potential as a biomarker associated with AMD. Given that MLR is a simple and cost-effective biomarker derived from routine blood tests, it may serve as a useful reference in AMD risk stratification and monitoring. Further research is needed to explore the underlying mechanisms and validate its clinical utility in diverse populations.

## Data Availability

The datasets generated and/or analyzed in this study are available from: NHANES database: [https://wwwn.cdc.gov/nchs/nhanes/], accessed October 2024.

## Abbreviations

AMD: Age-Related Macular Degeneration
AUC: Area Under the Curve
BMI: Body Mass Index
CI: Confidence Interval
CNV: Choroidal Neovascularization
CRP: C-Reactive Protein
MLR: Monocyte-to-Lymphocyte Ratio
NHANES: National Health and Nutrition Examination Survey
OR: Odds Ratio
PIR: Poverty-to-Income Ratio
RCS: Restricted Cubic Splines
ROC: Receiver Operating Characteristic
SD: Standard Deviation
wAMD: Wet-Type Age-Related Macular Degeneration
